# Salt Stress Induced Variation in DNA Methylation Pattern and Its Influence on Gene Expression in Contrasting Rice Genotypes

**DOI:** 10.1371/journal.pone.0040203

**Published:** 2012-06-28

**Authors:** Ratna Karan, Teresa DeLeon, Hanamareddy Biradar, Prasanta K. Subudhi

**Affiliations:** School of Plant, Environmental, and Soil Sciences, Louisiana State University Agricultural Center, Baton Rouge, Louisiana, United States of America; National Taiwan University, Taiwan

## Abstract

**Background:**

Salinity is a major environmental factor limiting productivity of crop plants including rice in which wide range of natural variability exists. Although recent evidences implicate epigenetic mechanisms for modulating the gene expression in plants under environmental stresses, epigenetic changes and their functional consequences under salinity stress in rice are underexplored. DNA methylation is one of the epigenetic mechanisms regulating gene expression in plant’s responses to environmental stresses. Better understanding of epigenetic regulation of plant growth and response to environmental stresses may create novel heritable variation for crop improvement.

**Methodology/Principal Findings:**

Methylation sensitive amplification polymorphism (MSAP) technique was used to assess the effect of salt stress on extent and patterns of DNA methylation in four genotypes of rice differing in the degree of salinity tolerance. Overall, the amount of DNA methylation was more in shoot compared to root and the contribution of fully methylated loci was always more than hemi-methylated loci. Sequencing of ten randomly selected MSAP fragments indicated gene-body specific DNA methylation of retrotransposons, stress responsive genes, and chromatin modification genes, distributed on different rice chromosomes. Bisulphite sequencing and quantitative RT-PCR analysis of selected MSAP loci showed that cytosine methylation changes under salinity as well as gene expression varied with genotypes and tissue types irrespective of the level of salinity tolerance of rice genotypes.

**Conclusions/Significance:**

The gene body methylation may have an important role in regulating gene expression in organ and genotype specific manner under salinity stress. Association between salt tolerance and methylation changes observed in some cases suggested that many methylation changes are not “directed”. The natural genetic variation for salt tolerance observed in rice germplasm may be independent of the extent and pattern of DNA methylation which may have been induced by abiotic stress followed by accumulation through the natural selection process.

## Introduction

Plant epigenetics has received considerable attention for both basic and applied research in recent years because understanding of the epigenetic regulation of plant growth and development could create new genetic variation for improving crop productivity as well as adaptation to stress environment [1]. Epigenetics refers to heritable variation in gene regulation resulting from covalent modifications of DNA and its associated chromatin proteins without changing the underlying nucleotide sequences [2]. Such epigenetic modifications are reversible and can alter the phenotypic appearance [3]. Cytosine methylation is a conserved epigenetic mechanism involved in many important biological processes, including transposon proliferation, genomic imprinting, and regulation of gene expression [4], [5]. It is usually associated with inactivation of genes while demethylation results in gene activation [6]. Genome-wide high-resolution mapping and functional analysis of DNA methylation in rice revealed that 8% of active genes were methylated within their promoter, while methylation within transcribed regions was observed in 31% of the expressed genes [5].

Due to erratic global climate, crop plants are frequently exposed to a variety of abiotic stresses including salinity resulting in reduced crop productivity. Analysis of stress related genes and their regulation of expression in response to abiotic stresses are commonly employed for enhanced understanding of the plants ability to adapt under abiotic stress environments. However, growing evidences implicate epigenetic mechanisms, such as DNA methylation and chromatin modification in regulating gene expression for plants’ responses to environmental stresses [7]. Demethylation of certain functionally inactive genes can occur due to exposure to abiotic stresses [8]. Abiotic stresses may cause heritable alterations in cytosine methylation by forming novel epialleles [9]. The expression of certain genes modified by epigenetic mechanism can be reverted to its earlier state and may show transgenerational inheritance [10]. Responses to the external stress vary among plant species; some of them can modulate their physiological and developmental machinery to mitigate the impact of stress whereas others are highly sensitive. Mangrove plants growing in contrasting natural habitats, riverside and salt marsh, differed with respect to cytosine methylation despite little genetic variation [11].

Methylation-sensitive amplified polymorphism (MSAP) is a powerful technique for studying the genome methylation status [12]. It is a modification of the AFLP technique in which isoschizomers, *Msp*I and *Hpa*II, are employed as ‘frequent-cutter’. Both *Msp*I and *Hpa*II recognize the same restriction site (5′-CCGG-3′), but show differential sensitivity to DNA methylation. This technique has been applied to evaluate the level and pattern of cytosine methylation in plants [13]. Effect of salinity stress on cytosine methylation variation has been studied in crop plants [14]–[15]. The epigenetic variation need to be explored in more genotypes so that useful variability can be identified and exploited in crop breeding programs to enhance crop adaptation in unfavorable environments.

Rice is one of the most salt-sensitive cereal crops and their sensitivity varies among different genotypes [16], [17]. Some rice genotypes possess unique ability to rapidly adapt to a toxic level of salt stress whereas others are highly susceptible indicating their uniqueness in genetic makeup and regulatory architecture. However, differences in methylation pattern and epigenetic response in contrasting rice varieties under salinity stress has been underexplored. In this study, we have investigated the extent and pattern of cytosine methylation under non-stress and salinity stress in four contrasting genotypes of rice using MSAP technique. Methylation and demethylation patterns were genotype and tissue specific irrespective of the degree of salt tolerance of individual rice genotype. MSAP fragments were localized onto different rice chromosomes and majority of these fragments showed homology with stress responsive genes, retrotransposons, and genes involved in chromatin modification. The differential expression patterns of stress related genes influenced by methylation or demethylation status under salinity stress in the rice genotypes revealed possible role of epigenetic mechanism in stress adaptation and the natural genetic variability in salinity tolerance phenotype may be independent of the extent and pattern of DNA methylation.

## Results

### Salinity Tolerance of Rice Genotypes

Scoring of visual salt stress injury using a modified standard evaluation system (SES) [18] after 12 days and 15 days of imposition of salt stress showed clear differences among rice genotypes. After 15 days of salt stress, most of Bengal, IR29 and Nipponbare plants were dead while Pokkali, Nonabokra, and Geumgangbyeo survived but showed salinity injury (Figure S1). Mean SES scores for IR29, Bengal, Nipponbare, Geumgangbyeo, Nonabokra, and Pokkali were 9.0, 8.8, 8.3, 4.3, 2.8, and 2.3, respectively. These scores indicated that Bengal and Nipponbare are highly salt sensitive like IR29, Geumgangbyeo is moderately tolerant, and Pokkali and Nonabokra are tolerant to salt stress. Comparison of uptake of Na^+^, K^+^, and K^+^/Na^+^ ratio (Figure S2) further confirmed the level of salt tolerance based on SES scores. The K^+^/Na^+^ ratios were higher in tolerant genotypes compared to susceptible genotypes under both control and salt stress.

### Extent and Pattern of DNA Methylation Under Control and Salinity Stress

**Figure 1:**
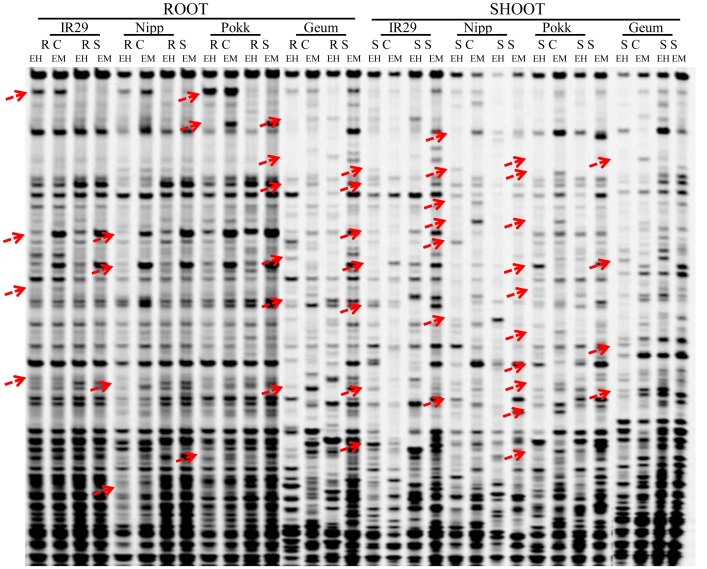
A representative MSAP gel using the primer combination *Eco*RI-ACG/*Msp*I-AATC. Both control and salinity stressed root and shoot of rice genotypes, IR29, Nipponbare (Nipp), Pokkali (Pokk), Geumgangbyeo (Geum) were used for MSAP analysis. EH and EM refer to digestion with *Eco*RI+*Hpa*II and *Eco*RI+*Msp*I, respectively. RC: root control; RS: root stress; SC: shoot control; SS: shoot stress.

Thirty two primer pair combinations were used to determine the cytosine methylation status in the root and shoot of four contrasting rice genotypes at seedling stage after 24 h of exposure to salinity stress and control (non-stress) conditions. Based on the presence or absence of bands (Figure 1), 1582–1635 clear and reproducible DNA fragments were amplified from root or shoot of each genotype of rice under control or salinity stress (Table 1). Under control condition in shoot, total methylation of CCGG sequences was 46.4% in Nipponbare, 48.6% in Geumgangbyeo, 57.2% in Pokkali, and 59.3% in IR29 while in root, total methylation ranged between 19.8% (Geumgangbyeo) and 32.6% (Nipponbare). In shoot, salinity stress decreased the percentage of total methylated bands in IR29 (from 59.3% to 51.7%) and Geumgangbyeo (from 48.6% to 34.5%) but increased in Nipponbare (from 46.4% to 57.6%) and Pokkali (from 57.2% to 65.3%) in comparison with the control indicating alteration of DNA methylation by salinity stress in different rice genotypes (Table 1A). However, in root, the number of fully methylated bands was reduced under salt stress compared with the control in all the four rice genotypes but it was more pronounced in Nipponbare (Table 1B). Interestingly, a general trend of higher level of fully methylated and hemimethylated bands was observed in shoot than root under both control and salinity stress in all four genotypes of rice. The fully methylated loci were always more than hemi-methylated loci (Table 1).

**Table 1:** DNA methylation changes in shoot and root at seedling stage under non-stress and salinity stress conditions.

	IR29		Nipponbare			Pokkali			Geumgangbyeo	
MSAP band type	Control	Salinity	Control	Salinity		Control		Salinity	Control	Salinity
A. Shoot										
I	665	789	877	692		698		566	838	1067
II	201	113	246	290		293		317	143	119
III	322	425	205	235		171		158	160	93
IV	446	308	306	418		471		593	489	350
Total amplified bands	1634	1635	1634	1635		1633		1634	1630	1629
MSAP (%)^a^	59.3	51.7	46.4	57.6		57.2		65.3	48.6	34.5
Fully methylated band (%)^b^	47.0	44.8	31.3	39.9		39.3		45.9	39.8	27.2
Hemi-methylated band (%)^c^	12.3	6.9	15.1	17.7		17.9		19.4	8.8	7.3
B. Root										
I	1276	1314	1103		1310		1231	1232	1311	1319
II	7	23	34		39		32	34	10	19
III	98	84	239		83		91	114	95	97
IV	254	211	259		203		232	202	218	197
Total amplified bands	1635	1632	1635		1635		1586	1582	1634	1632
MSAP (%)^a^	21.9	19.5	32.6		19.9		22.4	22.1	19.8	19.2
Fully methylated band (%)^b^	21.5	18.1	30.5		17.5		20.4	19.9	19.2	18.0
Hemi-methylated band (%)^c^	0.4	1.4	2.1		2.4		2.0	2.2	0.6	1.2

a MSAP (%)  =  [(II+III+IV)/(I+II+III+IV)]×100;

b Fully methylated bands (%) = [(III+IV)/(I+II+III+IV)]×100;

c Hemimethylated bands (%) = [(II)/(I+II+III+IV)]×100.

Type I indicated absence of methylation due to the presence of bands in both *EcoR*I/*Hpa*II and *Eco*RI/*Msp*I digest; type II bands appeared only in *EcoR*I/*Hpa*II digestion but not in the *Eco*RI/*Msp*I digest; type III generated bands obtained in *Eco*RI/*Msp*I digest but not in the *EcoR*I/*Hpa*II digest; and type IV represents the absence of band in both enzyme combinations.

### Salinity Induced Methylation/Demethylation Changes in Contrasting Genotypes of Rice

All possible banding patterns between control and salinity stress in shoot or root of the four rice genotypes were compared to find out the changes in cytosine methylation patterns under salinity stress. Sixteen different banding patterns between control and salinity stress were observed in the MSAP gels (Table 2). The pattern A-D represented monomorphic class in which methylation pattern was same in control and salinity stress treatment. The patterns E–J indicated cytosine demethylation patterns whereas possible cytosine methylation events induced by salt stress were represented by the patterns K-P. Around 44.9%–66.7% of the CCGG sites in shoot and 84.8%–90.9% in root remain unchanged under salinity stress (Table 2). The percentage of demethylated bands under salt stress was 29.7%, 17.5%, 11.5%, and 25.1% in shoot while it was 6.8%, 8.1%, 8.3%, and 6.4% in root of IR29, Nipponbare, Pokkali, and Geumgangbyeo, respectively, indicating relatively more DNA demethylation events in salt stressed shoot than in the root of all four rice genotypes (Table 2). In general, salinity stress induced more DNA demethylation and methylation in shoot of all four genotypes.

**Table 2:** Analysis of DNA methylation patterns under salinity stress with respect to control condition in the shoot and root of seedlings of rice varieties, IR29, Nipponbare (Nipp), Pokkali (Pokk), and Geumgangbyeo (Geum).

Description of Pattern	Class	Banding Pattern				Shoot				Root			
		Control		Salinity									
		*Hpa*II	*Msp*I	*Hpa*II	*Msp*I	IR29	Nipp	Pokk	Geum	IR29	Nipp	Pokk	Geum
	A	1	0	1	0	20	62	42	17	3	7	4	3
No change	B	0	1	0	1	108	44	30	12	54	55	54	51
	C	1	1	1	1	505	541	287	775	1238	1168	1131	1240
	D	0	0	0	0	184	180	373	282	188	131	153	153
	Total					817(50.0%)	827(50.6%)	732(44.9%)	1086(66.7%)	1483(90.9%)	1361(85.8%)	1342(84.8%)	1447(88.7%)
	E	1	0	1	1	62	70	17	80	3	17	21	5
	F	0	1	1	1	136	53	42	105	39	26	28	30
Demethylation	G	0	0	1	1	85	27	19	107	34	49	52	43
	H	0	1	1	0	25	37	30	16	3	7	3	4
	I	0	0	1	0	50	49	43	56	10	13	5	5
	J	0	0	0	1	127	50	36	44	22	16	22	17
	Total					485(29.7%)	286(17.5%)	187(11.5%)	408(25.1%)	111(6.8%)	128(8.1%)	131(8.3%)	104(6.4%)
	K	1	1	1	0	18	142	202	30	7	12	22	7
	L	1	1	0	1	113	98	75	16	7	7	38	29
Methylation	M	1	1	0	0	29	96	134	17	21	46	37	32
	N	1	0	0	1	77	43	17	21	1	5	0	0
	O	1	0	0	0	42	71	217	24	0	5	7	2
	P	0	1	0	0	53	71	68	27	2	21	5	10
	Total					332(20.3%)	521(31.9%)	713(43.6%)	135(8.2%)	38(2.3%)	96(6.1%)	109(6.9%)	80(4.9%)

A score of 1 and 0 represents presence and absence of bands, respectively. Values in parentheses indicate percentage of bands in each pattern which was determined by dividing number of bands in each pattern by total number of bands in all three patterns.

The test of independence between three different methylation patterns and salt treatments, control and salt stress conditions, was carried out using chi-square and likelihood ratio statistics (Table S1). The large value of chi-square test and likelihood ratio statistic as well as small *P*-value for both shoot and root methylation level in IR29 and Nipponbare (both salt susceptible) and only for shoot in Pokkali (tolerant) and Geumgangbyeo (moderately tolerant) suggested association between stress conditions and the level of methylation. IR29 and Geumgangbyeo showed reduced level of hemi- and full methylation whereas it was increased in Pokkali and Nipponbare in shoot under salt stress compared to control. In root, more loci were hemi methylated but fewer loci were fully methylated in response to salt stress.

### Analysis of the Differentially Methylated DNA Sequences

A random set of ten polymorphic fragments showing unique banding pattern in individual genotype for methylation/demethylation under salinity stress compared to control were sequenced to identify the nature of DNA sequences involved in methylation-demethylation under salinity (Table 3). The size of polymorphic DNA fragments varied between 60 to 324 bp and these fragments were distributed on the rice chromosomes 1, 2, 3, 4, and 11. The positions of cytosine alteration due to salinity stress were located in exon and UTR region of affected loci suggesting the gene-body specific methylation of MSAP loci. BLAST analysis of sequenced fragments revealed that the sequences are homologous to retrotransposons, peptidyl prolyl cis trans isomerase, calcineurin B, histone methyl transferase, rho guanine nucleotide exchange factor, cystathionine gamma synthase, DUF domain containing protein, methyl CG binding domain containing protein, and pentatricopeptide repeat domain containing protein (Table 3).These results suggest that the genes involved in wide range of cellular functions were affected mostly by gene-body specific cytosine methylation-demethylation due to salinity stress in rice.

**Table 3:** BLAST results of ten randomly selected polymorphic methylated fragments and their location on rice genome.

MSAPfragment	Methylation status under stress	Tissuetype	Genotype	Gene	TIGR Locus ID	E value	Position
P1	Methylated	shoot	IR29	Peptidyl-prolyl cis-transisomerase, FKBP-type	Os02g52290	8.3e-24	3′ UTR
P2	Methylated	shoot	Geumgangbyeo	Calcineurin B	Os01g39770	2.9e-41	3′ UTR
P3	Demethylated	shoot	IR29	Cystathionine gamma-synthase	Os03g25940	1.8e-07	Exon
P4	Methylated	shoot	IR29	Rho guanine nucleotideexchange factor	Os02g44330	2.1e-19	Exon
P5	Demethylated	shoot	IR29	DUF1230 domain containing protein	Os03g32490	7.3e-60	Exon
P6	Demethylated	shoot	IR29	Methyl-CpG domain containing protein	Os04g19684	2.3e-29	Exon
P7	Methylated	shoot	IR29	Histone-lysineN-methyltransferase	Os01g70220	8.9e-56	5′ UTR
P8	Demethylated	shoot	Nipponbare	Retrotransposon protein	Os11g23900	3.7e-56	Exon
P9	Demethylated	shoot	IR29	Retrotransposon protein,Ty1-copia subclass	Os01g60309	2.5e-40	Exon
P10	Demethylated	shoot	IR29	Pentatricopeptide repeatdomain containing protein	Os11g37330	8.9e-48	Exon

### Expression Analysis of MSAP loci in Root and Shoot of Rice Genotypes

**Figure 2:**
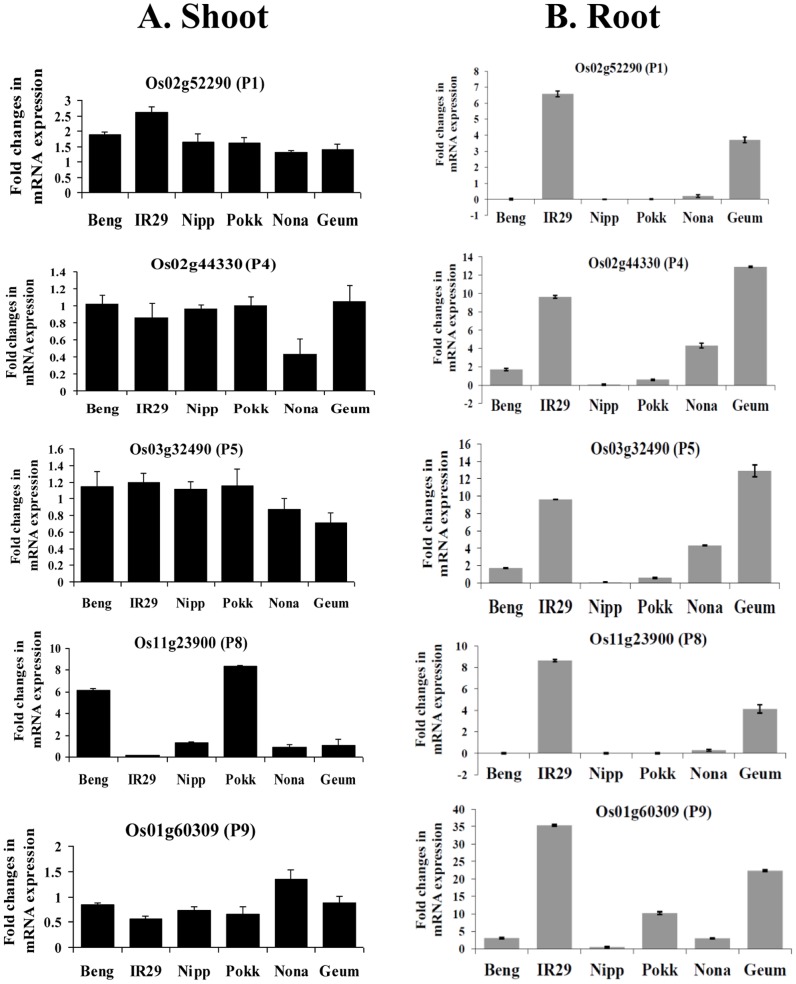
Expression profiles of MSAP loci in rice under salt stress. Salinity stressed shoot (A) and root (B) of six genotypes of rice, Bengal (Beng), IR29, Nipponbare (Nipp), Pokkali (Pokk), Nonabokra (Nona), and Geumgangbyeo (Geum) were used for quantifying the expression of MSAP loci relative to control. Real-time PCR analysis was performed using gene-specific primers. The expression of each gene in different RNA samples was normalized with the expression of internal control gene, rice elongation factor 1 α (*eEF1* α). Fold changes in mRNA expression for each candidate gene in different genotypes were calculated relative to its control using ddCt method. The values represented are the mean of two biological replicates, each with three technical replicates. Error bars indicate the standard deviation.

Expression analysis of five MSAP polymorphic genes was done using quantitative RT-PCR in shoot and root of six contrasting rice genotypes under control and salinity stress to determine the effect of methylation changes on the gene expression (Figure 2). Gene Os02g44330 (P4), methylated in the shoot of IR29, showed downregulation under salinity stress in shoot of IR29 and Nonabokra, whereas its expression remain unchanged in the shoot of the remaining four genotypes under salinity stress in comparison to control (Figure 2A). However in the root, its expression was enhanced in IR29, Nonabokra, and Geumgangbyeo but downregulated in Nipponbare and Pokkali. Bengal showed a slight increase in expression. Similarly, expression changes of Os03g32490 (P5) which was demethylated in the shoot of IR29, was minimal with increased expression in the shoot of IR29, Bengal, Nipponbare, and Pokkali, but reduced expression in Nonabokra and Geumgangbyeo under salinity stress (Figure 2A). In roots of IR29 and Geumgangbyeo, salt stress enhanced the expression of P5 but reverse was the case in Bengal, Nipponbare, Pokkali, and Geumgangbyeo (Figure 2B). One of the demethylated gene, Os11g23900 (P8) in the shoot of Nipponbare, showed 6–8 fold increase expression in shoot of Bengal, and Pokkali but was downregulated in IR29, while remain unchanged in Nipponbare, Nonabokra, and Geumgangbyeo under salinity (Figure 2A). It was upregulated in roots of IR29 and Geumgangbyeo but down regulated in other genotypes (Figure 2B).The gene Os02g52290 (P1) which was methylated in shoot of IR29 and homologous to peptidyl-prolyl cis-trans isomerase, showed increased expression in the shoot and root of IR29 and Geumgangbyeo under salinity stress. The expression of P1 was enhanced in the shoot of other four genotypes under salinity stress than control, but was downregulated in the root of Bengal, Nipponbare, Pokkali, and Nonabokra. Another gene, Os01g60309 (P9), a retrotransposon, demethylated in the shoot of IR29, showed downregulation in shoot of IR29, Bengal, Nipponbare, Pokkali, and Geumgangbyeo whereas it was upregulated in Nonabokra. In root, expression of P9 was enhanced under salinity stress in IR29, Pokkali, and Geumgangbyeo, but remained unchanged or slightly increased in Nipponbare, Bengal, and Nonabokra. Therefore, the methylation pattern within the gene-body region did not always correspond to its expression changes under salinity stress and expression variation was genotype specific.

### Bisulfite Sequencing of MSAP Loci

Bisulfite sequencing of two selected MSAP loci, Os02g52290 (P1) and Os01g60309 (P9) was conducted to assess the cytosine methylation status. The bisulfite nucleotide sequences from top strands of these loci were similar with their corresponding loci from TIGR database and most of the methylated cytosines belonged to CG types (Figures S3, S4). Alignment of Os02g52290 (P1) (Figures S3 and S5) sequence with the bisulfite treated DNA sequences from root and shoot of all the six rice genotypes under non-stress and salinity stress conditions revealed that methylated cytosine present under non-stress conditions at position 3, 5, and 29, did not change even under salinity stress. At 75^th^ nucleotide position, cytosine gets demethylated under salinity stress in shoot and remained unmethylated under both conditions in root in Pokkali while it maintained methylation status in other genotypes. Cytosine at position 154 was only methylated in shoot of Bengal under non-stress condition. The patterns of methylation were identical at both 234 and 243 positions. In Bengal, cytosine at position 264 was demethylated in shoot and methylated in root, whereas, it maintained its methylated or unmethylated status in rest five genotypes of rice under salinity compared to non-stress condition. At position 278, cytosine maintained methylated status in Nipponbare in both tissues under control or salinity stress. Cytosine methylation (%) was reduced in shoot under salt stress compared with control in Bengal, Nipponbare, and Pokkali, but was increased IR29 and remained unchanged in Nonabokra and Geumgangbyeo (Figure S3). Furthermore, cytosine methylation in root under salinity was slightly increased in Bengal, but remained unchanged in IR29, Nipponbare, Pokkali, Nonabokra, and Geumgangbyeo.

In case of Os01g60309 (P9) (Figures S4 and S6), cytosines at 108 and 113 positions remained methylated under both conditions and both tissues in all genotypes but cytosine at 242 was methylated under salinity compared to control in all the genotypes except shoot of Nipponbare and root of IR29. Differential methylation pattern was observed in both tissues at 107^th^ position. At position 315, cytosine was methylated in shoot and root of all genotypes under both conditions, except in root of Geumgangbyeo and Nonabokra under salinity stress. Cytosine methylation (%) in shoot and root under salinity compared to control remained unaltered in Bengal, Nipponbare, and Nonabokra, increased in IR9, and decreased in Pokkali (Figure S4). However, cytosine methylation decreased in shoot and did not change in root under salinity in comparison to control in Geumgangbyeo. Although salinity affected the methylation status of cytosines in these two selected loci, the changes were genotype and organ specific irrespective of salinity tolerance of rice genotypes.

## Discussion

Evidences are growing in favor of epigenetic modifications influencing plants’ preparedness for stress adaptation through regulation of gene expression [19], [20]. However, the relationship between DNA methylation and abiotic stress tolerance was rarely studied in important field crops [15]. In this study, we assessed the epigenetic consequences of salinity stress in rice genotypes with contrasting salt tolerance behavior. Among the rice genotypes used in this study, Pokkali and Nonabokra were tolerant and IR29 was highly salt sensitive [16], [17]. Bengal and Nipponbare showed salt sensitivity whereas Geumgangbyeo showed moderate tolerance (Figures S1 and S2).

We employed MSAP technique to assess the extent and pattern of DNA methylation in response to salt stress in both shoot and root of rice. Overall, the amount of DNA methylation was more in shoot compared to root (Table 1), indicating unique biological functions performed by each tissue in response to salt stress. Since root experience the stress first, hypomethylation suggests the preparedness of more stress responsive genes to respond to salt stress. Hypomethylation has been reported to affect plant development [21]. We did not observe any methylation pattern specific to salt tolerant or salt susceptible genotypes in root or shoot under salt stress (Table 2). However, significant association between salt treatment and level of methylation was seen in shoot of all four genotypes and in root of only IR29 and Nipponbare (Table S1), suggesting that many methylation changes are not “directed”. Genotype specific DNA methylation has been reported in rice [13]. Furthermore, epigenetic diversity created through methylation changes might be responsible for inter-cultivar differences [21] but there was no correlation between genetic distance and DNA methylation polymorphism [13], [22]. Since these methylation changes have functional consequences and heritable epigenetic changes can be induced by abiotic stresses [23], [24], such changes might have been subjected to selection resulting in intervarietal differentiation and variation in methylation pattern among the genotypes as reported here. Therefore, DNA methylation polymorphism may be helpful for the understanding of genetic and functional differences among rice cultivars and genotype specific polymorphism observed in this study may be exploited for generating epigenetic markers for DNA fingerprinting and molecular breeding.

Salinity stress causes major alteration in the expression pattern of genes involved in diverse physiological and regulatory pathways in contrasting genotypes of rice at different stages of development [25], [26]. Despite the sequencing of limited number of MSAP fragments, we could identify three different categories of genes such as retrotransposons, abiotic stress responsive genes, and genes involved in chromatin modification (Table 3). The use of isoschizomers thus targeted the gene-rich areas spread over of the genome as expected (Table 3) [27]. The genes, peptidyl-prolyl cis-trans-isomerase FKBP77, calcinurin B, and guanine nucleotide binding proteins of the Rho family are involved in abiotic stress response, ABA signaling, and signal transduction in plants [28]–[30] where as methyl CG binding domain containing proteins, histone methyl transferase, and cystathionine γ-synthas are implicated in chromatic remodeling and DNA methylation [31]–[33]. The pentatricopeptide repeat (PPR) protein is involved in the posttranscriptional processes of gene expression in plant organelles [34]. Identification of diverse category of genes with altered DNA methylation pattern in our study provides clear evidence that epigenetic modification plays a critical role in plants adaptation to environmental challenges [20].

Retrotransposons are triggered in response to salt stress and may generate true genetic or epigenetic changes thus increasing plants’ adaptation to abiotic stresses [35]. Plant retrotransposons are usually hypermethylated in comparison with host genes and are said to be epigenetically silenced [36]. Both retrotransposon genes identified in our study were demethylated in response to salt stress and the expression level was genotype specific. Such variation in the methylation status in different tissue types has been reported in earlier studies involving LTR (Long Terminal Repeat) retrotransposons in rice [37]. Therefore, the elucidation of the epigenetic control of transcription and transposition of retrotransposons may provide a novel strategy to create genetic variation for developing stress tolerant crop plants in future [1].

It is not yet clear if the DNA methylation is directed at specific sequences or a random response to abiotic stresses. Few genes such as peptidyl prolyl cis-trans isomerase, histone lysine N-methyl transferase, and retrotransposons differentially methylated under salt stress in our study were also reported to be polymorphic under salt stress in another MSAP study in rice [15]. This observation suggested preferential methylation of some category of genes under abiotic stress [38].

Despite the widely held belief that DNA methylation is associated with transcription inhibition [39], [40], weak relationship between the hypermethylation status of the genes and their expression level has been reported [41]. Around 33% of the genes in *A. thaliana* were methylated within transcribed region and but were highly expressed and constitutively active [42]. Analysis of the methylation status of two selected MSAP loci, Os02g52290 (P1) and Os01g60309 (P9) by bisulphite sequencing revealed organ and genotype specific alteration in cytosine methylation under both control and salinity stress (Figures S3, S4). In case of Os02g52290 (P1), gene expression under salinity was inversely related to cytosine methylation in only shoot of all genotypes except IR29 (Figure 2). But no such relationship could be evident for Os01g60309 (P9). Although tissue specific differences in cytosine methylation has been recently reported in sorghum, only few of the tissue-specific differentially methylated regions could be correlated with its tissue specific expression [43].

In *A. thaliana* moderately transcribed genes showed highest methylation level within gene bodies, whereas genes with expression levels at either extreme are less likely to be methylated [40]. Gene body methylation may improve the accuracy of splicing and promoter expression and may be functionally important than unmethylated genes [44]. The gene body methylation reported for all the MSAP loci suggests it may have an important role in regulating gene expression in organ and genotype specific manner under salinity stress which is in agreement with earlier studies [15], [43], [45].

Our results suggest that the tissue and genotype specific epigenetic changes in the rice genome may be an important alternative regulatory mechanism for sensing and responding to the salt stress through modification of the expression network of salt stress responsive genes as well as the genes involved in epigenetic modification. This investigation further suggests that evolution of natural genetic variability for salinity tolerance in rice germplasm may be independent of the extent and pattern of DNA methylation in rice.

## Materials and Methods

### Plant Materials and Stress Treatment

Six rice genotypes (Bengal, IR29, Nipponbare, Pokkali, Nona Bokra and Geumgangbyeo) were used in this investigation. Pokkali (IRRI Acc No. IRGC 108921), Nonabokra (IRRI Acc. No. IRGC 22710), IR29 (Acc No. IRGC 30412) were procured from the International Rice Research Institute, Philippines. Geumgangbyeo (PI 464588) is a South Korean semi-dwarf variety listed as the most salt tolerant rice variety at seedling stage (salt tolerance value of 5.6) in the USDA- Germplasm Resources Information Network (GRIN) database. Bengal is a semi-dwarf, early maturing medium-grain rice cultivar released by the Rice Research Station of the Louisiana Agricultural Experiment Station in 1992. Nipponbare (GSOR #70) was obtained from the Dale Bumpers National Rice Research Center located at Sturtgart, AR, USA. Two salt tolerant rice genotypes (Pokkali and Geumgangbyeo) and two salt susceptible cultivars (IR29 and Nipponbare) were used for MSAP analysis. All six rice genotypes were used for gene expression study. Seeds of all the genotypes were germinated in two different hydroponic trays and grown under 16 h light and 8 h dark condition in Yoshida nutrient solution [46]. After fifteen days, seedlings in one tray were provided with Yoshida solution containing150 mM NaCl whereas seedlings in the other tray were maintained in normal Yoshida solution. Visual salt stress injury was scored using a modified standard evaluation system (SES) 12 days and 15 days after salt stress [18]. SES scores were given on a 1–9 scale where 1 (highly tolerant)  =  normal growth, 2 (tolerant)  =  nearly normal growth, but leaf tips or few leaves whitish and rolled, 3 (moderately tolerant)  =  growth severely retarded, most leaves rolled and only few are elongating, 7 (susceptible)  =  complete cessation of growth, most leaves dry and some plants drying, and score 9 (highly susceptible)  =  almost all plants dead or dying. Roots and shoots of both control and salt stressed seedlings were harvested separately after 24 h of salinity stress and immediately frozen in liquid nitrogen and stored at −80°C freezer till further use.

To estimate the Na^+^, K^+^ in six genotypes of rice, fifteen day old hydroponically grown seedlings were supplied with 150 mM of NaCl. After 7 days of salt stress, unstressed and stressed shoot tissues were harvested. One hundred milligrams of fresh shoot tissues were digested with 0.1% of HNO_3_ at 100°C for 45 min and then Na^+^ and K^+^ concentrations were measured from three replicates of each genotype using inductively coupled plasma-mass spectrometry (ICP-MS, Perkin-Elmer Plasma 400 emission spectrometer).

### DNA Extraction and Methylation Sensitive Amplified Polymorphism (MSAP) Analysis

Genomic DNA from root and shoot were isolated by method described by Murray and Thompson [47]. MSAP technique was employed following the procedure of Subudhi et al. [48] with little modification, using a pair of methylation-sensitive restriction enzymes, *Msp*I and *Hpa*II in combination with Eco*RI*. The adapter, preamplification, and selective amplification primers were listed in table S2. In this study, we used 700 and 800 fluorescently labeled *Eco*RI primers during selective amplification step. The denatured PCR-amplified products were separated on 6% denaturing polyacrylamide gels on a LiCor 4300 DNA Analyzer (LiCOR Inc., Lincoln, NE) at 1000 V for 2.5 h. Electrophoregrams of all AFLP profiles were visually scored for polymorphisms for the presence (1) or absence (0) of fragments ranging from 60 bp to 350 bp in a binary matrix. Regular polyacrylamide gels were run in a BioRad Sequi-Gen unit ® (38×50 cm) using the selected primer combinations showing changes in methylation/demethylation pattern using non labeled *Eco*RI and *Hpa*II/*Msp*I primer pairs followed by silver staining for visualization and elution of desired band.

The amplified DNA fragments were divided into four types based on presence or absence of bands due to the differential sensitivity of *Msp*I and *Hpa*II restriction digestion. Type I represents the presence of bands in both enzymes combinations i.e. *EcoR*I/*Hpa*II and *Eco*RI/*Msp*I, type II bands appeared only in *EcoR*I/*Hpa*II but not in the *Eco*RI/*Msp*I, type III generated bands in *Eco*RI/*Msp*I but not in the *EcoR*I/*Hpa*II, and type IV represents the absence of band in both enzyme combinations. Type II indicates the hemimethylated state of DNA due to methylation in one DNA strand but not in its complementary strand [49]. Type III represents the case of full CG (internal cytosine) methylation, whereas type IV is the case of full methylation at both cytosines. Percentage of polymorphic MSAP bands in table 1 was calculated using the following formula:
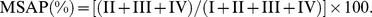



### DNA Sequencing and Homology Search of Differentially Expressed MSAP Fragments

DNA bands showing desirable polymorphism were excised from silver stained AFLP gel. DNA was eluted from the excised bands by adding 30 µl of deionized sterile water followed by heating at 100°C for 5 min. Eluted DNA was then reamplified using corresponding primer pairs and DNA band of desired size was excised from agarose gel and eluted using gel elution column (Qiagen, Valencia, USA). Eluted bands were sequenced at the Gene Lab of the School of Veterinary Medicine, Louisiana State University. Identity of obtained DNA sequence was confirmed by nucleotide-nucleotide homology search and the position of MSAP fragment within the rice gene and chromosome location was determined by alignment with homologous gene sequences in the rice genome database available at the Rice Genome Annotation Project Website (http://rice.plantbiology.msu.edu/).

### RNA Isolation and Expression Analysis

Total RNA was isolated from both control and salt stressed tissues using total RNA isolation kit (Qiagen, USA). Reverse transcription for the synthesis of 1^st^ strand cDNA was carried out using single strand cDNA synthesis kit (BioRad, Hercules, USA) containing 2 µg of total RNA in a reaction volume of 20 µl. Primers for expression analysis of methylated genes in rice genotype were designed using Primer3 and manually verified by Oligoanalyzer (IDT Inc., USA) (Table S3). The transcript levels in different RNA samples were quantified by real-time PCR analysis employing MyiQ™ Real-Time PCR detection system (Bio-Rad, Hercules, USA). Diluted cDNA samples were used as template and mixed with 200 nM of each primer and SYBR Green PCR Master Mix (BioRad, Hercules, USA). PCR reactions were performed using the following parameters: 10 min at 95°C, 40 cycles of 15 s at 95°C and 1 min at 60°C in 96-well optical reaction plates. The identities of the amplified DNA and the specificity of the reaction were verified by agarose gel electrophoresis and melting curve analysis, respectively. Rice endogenous gene, *eEF1* α (elongation factor 1α) was used to normalize variance in the quality of RNA and the amount of input cDNA. The relative mRNA levels for each of the genes in different RNA samples were computed by ddCt method [50]. At least two different RNA isolations and synthesized cDNA were used for quantification and each cDNA sample was subjected to real-time PCR in triplicate.

### Bisulfite Sequencing

One micrograms of total DNA extracted from unstressed and salinity stressed tissues of six rice genotypes, collected 24 hours after imposition of salt stress, was modified by sodium bisulfite using Epitect Plus DNA Bisulfite Kit (Qiagen, USA) following the manufacturer’s protocol. An aliquot of 2 µl of bisulfite-treated DNA was used for each PCR reaction (25 µl) using EpiMark® Hot Start Taq DNA Polymerase (New England Biolab, USA). The primers for two MSAP loci corresponding to fragments, Os02g52290 (P1) and Os01g60309 (P9), were designed using Methyl Primer Express Software^R^ (Applied Biosystems) to amplify bisulfite-converted genomic DNA. The primers were: 5′ TGTAAGTTTGTTTTTGGTTTG-3′ (P1F), 5′-TACAACCCAAAACTTATTATC-3′ (P1R), 5′-ATTAAGGGTTGGTGTTATTTT-3′ (P9F), 5-CATCAAACCTTTTTCTTATAACTCC-3′(P9R). PCR amplified fragments were eluted from gel using QiaQuick Gel Extraction Kit (Qiagen, USA) and top strands of DNA were sequenced at the High Throughput Genomics Unit of the University of Washington, Seattle, USA, using gene specific forward primers. Methylation status of DNA was obtained by comparing the sequence of the bisulfite-treated DNA with that of untreated DNA, where conversion of a C to T indicated non-methylated C. In contrast, the absence of C to T conversion indicated methylation. Cytosine methylation status in the top strand of obtained nucleotide sequences were calculated using CyMATE v2 [51]. The methylation level for each of the three kinds of cytosines, CG, CHG, and CHH, (H represents A, T, or C) was calculated using following formula:

Methylated cytosine (%)  =  [Number of non-converted (methylated) cytosines/Total number of cytosines of each type]×100.

### Statistical Analyses

Chi-square test was used to test the independence between methylation level and salt stress condition using SAS Version 9.1 (*S*AS Institute, Cary, NC).

## Supporting Information

Figure S1 **Phenotype of rice genotypes after 15 days of salinity stress (150 mM NaCl).** Individual plants were scored for visual salt injury on a scale of 1–9 using the modified Standard Evaluation System [18].
(TIF)

Figure S2 **Effect of 150 mM NaCl on the K^+^/Na^+^ ratio in the shoot of six rice genotypes.** The rice genotypes are Bengal (Beng), IR29, Nipponbare (Nipp), Pokkali (Pokk), Nonabokra (Nona), and Geumgangbyeo (Geum). Each value is the mean ± standard error from three independent replicates after exposure of 15 day old seedlings to salt stress for 7 days.
(TIF)

Figure S3 **Bisulfite sequencing analysis of the locus Os02g52290 (P1).** Top strand of Os02g52290 was sequenced from the shoot and root of six genotypes of rice, Bengal (Beng), IR29, Nipponbare (Nipp), Pokkali (Pokk), Nonabokra (Nona), and Geumgangbyeo (Geum) under non-stress and salinity stress (150 mM NaCl for 24 h). Cytosine methylation count was given for three types of methylation, CH, CHG, and CHH (H: A, T or C). C: non-stress; S: 150 mM salinity stress; % mC: percentage of methylated cytosines.
(TIF)

Figure S4 **Bisulfite sequencing analysis of the locus Os01g60309 (P9).** Top strand of Os01g60309 was sequenced from the shoot and root of six genotypes of rice, Bengal (Beng), IR29, Nipponbare (Nipp), Pokkali (Pokk), Nonabokra (Nona), and Geumgangbyeo (Geum), under non-stress and salinity stress (150 mM NaCl for 24 h). Cytosine methylation count was given for three types of methylation, CH, CHG, and CHH (H: A, T or C). C: non-stress; S: 150 mM salinity stress; % mC: percentage of methylated cytosines.
(TIF)

Figure S5 **Sequence alignment of sodium-bisulfite-modified DNA of the locus Os02g52290 (P1).** Top strand of Os02g52290 was sequenced from the shoot and root of six genotypes of rice, Bengal (Beng), IR29, Nipponbare (Nipp), Pokkali (Pokk), Nonabokra (Nona), and Geumgangbyeo (Geum) under non-stress and salinity stress (150 mM NaCl for 24 h. Unmodified represents top strand of unmodified sequence of Os02g52290 from Nipoonbare. C: non-stress; S: salinity stress.
(TIF)

Figure S6 **Sequence alignment of sodium-bisulfite-modified DNA of the locus Os01g60309 (P9).** Top strand of Os02g52290 was sequenced from the shoot and root of six genotypes of rice, Bengal (Beng), IR29, Nipponbare (Nipp), Pokkali (Pokk), Nonabokra (Nona), and Geumgangbyeo (Geum) under non-stress and salinity stress (150 mM NaCl for 24 h). Unmodified represents top strand of unmodified sequence of Os01g60309 from Nipoonbare. C: non-stress; S: salinity stress.
(TIF)

Table S1 **Chi-square test and adjusted residuals for testing independence between methylation level and salt stress condition.**
(DOCX)

Table S2 **Adapters and primers sequences used for methylation sensitive amplified polymorphism analysis.**
(DOCX)

Table S3 **Primers used for quantitative reverse transcription polymerase chain reaction.**
(DOCX)

## References

[1] Mirouze M, Paszkowski J (2011). Epigenetic contribution to stress adaptation in plants.. Curr Opin Plant Biol.

[2] Fazzari MJ, Greally JM (2004). Epigenomics: beyond CpG islands.. Nat Rev Genet.

[3] Suzuki MM, Bird A (2008). DNA methylation landscapes: provocative insights from epigenomics.. Nat Rev Genet.

[4] Bird A (2002). DNA methylation patterns and epigenetic memory.. Genes Dev.

[5] Yan HH, Kikuchi S, Neumann P, Zhang WL, Wu YF (2010). Genome-wide mapping of cytosine methylation revealed dynamic DNA methylation patterns associated with genes and centromeres in rice.. Plant J.

[6] Zemach A, McDaniel IE, Silva P, Zilberman D (2010). Genome-wide evolutionary analysis of eukaryotic DNA methylation.. Science.

[7] Boyko A, Kovalchuk I (2011). Genome instability and epigenetic modification -heritable responses to environmental stress?. Curr Opin Plant Biol.

[8] Choi CS, Sano H (2007). Abiotic-stress induces demethylation and transcriptional activation of a gene encoding a glycerophosphodiesterase-like protein in tobacco plants.. Mol Genet Genomics.

[9] Kou HP, Li Y, Song XX, Ou XF, Xing SC (2011). Heritable alteration in DNA methylation induced by nitrogen-deficiency stress accompanies enhanced tolerance by progenies to the stress in rice (*Oryza sativa* L.).. J Plant Physiol.

[10] Bender J (2004). DNA methylation and epigenetics.. Annu Rev Plant Biol.

[11] Lira-Medeiros CF, Parisod C, Fernandes RA, Mata CS, Cardoso MA (2010). Epigenetic variation in mangrove plants occurring in contrasting natural environment.. PLoS One.

[12] Reyna-Lopez GE, Simpson J, Ruiz-Herrera J (1997). Differences in DNA methylation patterns are detectable during the dimorphic transition of fungi by amplification of restriction polymorphism.. Mol Genet Genomics.

[13] Ashikawa I (2001). Surveying CpG methylation at 5′-CCGG in the genomes of rice cultivars.. Plant Mol Biol.

[14] Zhong L, Xu YH, Wang JB (2009). DNA-methylation changes induced by salt stress in wheat *Triticum aestivum*.. Afr J Biotechnol.

[15] Wang WS, Zhao X, Pan Y, Zhu L, Fu B (2011). DNA methylation changes detected by methylation-sensitive amplified polymorphism in two contrasting rice genotypes under salt stress.. J Genet Genomics.

[16] Lee KS, Choi WY, Ko JC, Kim TS, Gregorio GB (2003). Salinity tolerance of *japonica* and *indica* rice (*Oryza sativa* L.) at the seedling stage.. Planta.

[17] Ismail AM, Heuer S, Thomson MJ, Wissuwa M (2007). Genetic and genomic approaches to develop rice germplasm for problem soils.. Plant Mol Biol.

[18] Gregorio GB, Senadhira D, Mendoza RD (1997). Screening rice for salinity tolerance.. IRRI Discussion Paper Series, no. 22, International Rice Research Institute, Manila, Philippines.

[19] Lukens LN, Zhan S (2007). The plant genome’s methylation status and response to stress: implications for plant improvement.. Curr Opin Plant Biol.

[20] Lopez-Maury L, Marguerat S, Bahler J (2008). Tuning gene expression to changing environments: from rapid responses to evolutionary adaptation.. Nat Rev Genet.

[21] Kakutani T (2002). Epi-alleles in plants: inheritance of epigenetic information over generations.. Plant Cell Physiol.

[22] Takata M, Yuji K, Yoshio S (2005). DNA methylation polymorphisms in rice and wild rice strains: detection of epigenetic markers.. Breed Sci.

[23] Steward N, Ito M, Yamaguchi Y, Koizumi N, Sano H (2002). Periodic DNA methylation in maize nucleosomes and demethylation by environmental stress.. J Biol Chem.

[24] Hashida S, Kitamura K, Mikami T, Kishima Y (2003). Temperature shift coordinately changes the activity and the methylation state of transposon *Tam3* in *Antirrhinum majus*.. Plant Physiol.

[25] Walia H, Wilson C, Condamine P, Liu X, Ismail AM (2005). Comparative transcriptional profiling of two contrasting rice genotypes under salinity stress during the vegetative growth stage.. Plant Physiol.

[26] Walia H, Wilson C, Zeng L, Ismail AM, Condamine P (2007). Genome-wide transcriptional analysis of salinity stressed *japonica* and *indica* rice genotypes during panicle initiation stage.. Plant Mol Biol.

[27] Tran R, Henikoff JG, Zilberman D, Ditt RF, Jacobsen S (2005). DNA methylation profiling identifies CG methylation clusters in *Arabidopsis* genes.. Curr Biol.

[28] Kurek I, Aviezer K, Erel N, Herman E, Breiman A (1999). The wheat peptidyl prolyl cis-trans-isomerase FKBP77 is heat induced and developmentally regulated.. Plant Physiol.

[29] Pandey GK, Cheong YH, Kim KN, Grant JJ, Li L (2004). The Calcium sensor calcineurin b-like 9 modulates abscisic acid sensitivity and biosynthesis in *Arabidopsis*.. The Plant Cell.

[30] Berken A (2006). ROPs in the spotlight of plant signal transduction.. Cell Mol Life Sci.

[31] Zemach A, Grafi G (2007). Methyl-CpG-binding domain proteins in plants: interpreters of DNA methylation.. Trends Plant Sci.

[32] Berr A, Xu L, Gao J, Cognat V, Steinmetz A (2009). *SET DOMAIN GROUP_25_* encodes a histone methyltransferase and is involved in *FLOWERING LOCUS C* activation and repression of flowering.. Plant Physiol.

[33] Kim J, Lee M, Chalam R, Martin MN, Leustek T (2002). Constitutive overexpression of cystathionine γ-synthase in *Arabidopsis* leads to accumulation of soluble methionine and S-methylmethionine.. Plant Physiol.

[34] Okuda K, Myouga F, Motohashi R, Shinozaki K, Shikanai T (2007). Conserved domain structure of pentatricopeptide repeat proteins involved in chloroplast RNA editing.. Proc Natl Acad Sci USA.

[35] Reinders J, Wulff BBH, Mirouze M, Marı ´-Ordonez A, Dapp M (2009). Compromised stability of DNA methylation and transposon immobilization in mosaic *Arabidopsis* epigenomes.. Genes Dev.

[36] Rabinowicz PD, Palmer LE, May BP, Hemann MT, Lowe SW (2003). Genes and transposons are differentially methylated in plants, but not in mammals.. Genome Res.

[37] Kashkush K, Khasdan V (2007). Large-scale survey of cytosine methylation of retrotransposons and the impact of readout transcription from long terminal repeats on expression of adjacent rice genes.. Genetics.

[38] Labra M, Ghiani A, Citterio S, Sgorbati S, Sala F (2002). Analysis of cytosine methylation pattern in response to water deficit in pea root tips.. Plant Biol.

[39] Bilichak A, Ilnystkyy Y, Hollunder J, Kovalchuk I (2012). The progeny of *Arabidopsis thaliana* plants exposed to salt exhibit changes in DNA methylation, histone modifications and gene expression.. PLoS One.

[40] Zilberman D, Gehring M, Tran RK, Ballinger T, Henikoff S (2007). Genome-wide analysis of *Arabidopsis thaliana* DNA methylation uncovers an interdependence between methylation and transcription.. Nat Genet.

[41] Vaillant I, Schubert I, Tourmente S, Mathieu O (2006). MOM1 mediates DNA-methylation-independent silencing of repetitive sequences in *Arabidopsis*.. EMBO Rep.

[42] Zhang X, Yazaki J, Sundaresan A, Cokus S, Chan SWL (2006). Genome-wide high-resolution mapping and functional analysis of DNA methylation in *Arabidopsis*.. Cell.

[43] Zhang M, Xu C, Wettstein DV, Liu B (2011). Tissue-specific differences in cytosine methylation and their association with differential gene expression in sorghum.. Plant Physiol.

[44] Takuno S, Gaut BS (2012). Body-methylated genes in *Arabidopsis thaliana* are functionally important and evolve slowly.. Mol Biol Evol.

[45] Wang WS, Pan YJ, Zhao XQ, Dwivedi D, Zhu LH (2011). Drought-induced site-specific DNA methylation and its association with drought tolerance in rice (*Oryza sativa* L.).. J Exp Bot.

[46] Yoshida S, Forno DA, Cook JH, Gomes KA (1976). Routine procedure for growing rice plants in culture solution. Laboratory manual for physiological studies of rice..

[47] Murray HG, Thompson WF (1980). Rapid isolation of high molecular weight plant DNA.. Nucl Acids Res.

[48] Subudhi PK, Nandi S, Casal C, Virmani SS, Huang N (1998). Classification of rice germplasm: III. High resolution fingerprinting of cytoplasmic genetic male sterile (CMS) lines with AFLP.. Theor Appl Genet.

[49] McClelland M, Nelson M, Raschke E (1994). Effect of site specific modification restriction endonucleases and DNA modification methyltransferases.. Nucleic Acids Res.

[50] Karan R, Singla-Pareek SL, Pareek A (2009). Histidine kinase and response regulator genes as they relate to salinity tolerance in rice.. Funct Integ Genomics.

[51] Hetzl J, Foerster AM, Raidl G, Mittelsten Scheid O (2007). CyMATE: a new tool for methylation analysis of plant genomic DNA after bisulphite sequencing.. Plant J.

