# Supplementary figures and images for: Salt Stress Induced Variation in DNA Methylation Pattern and Its Influence on Gene Expression in Contrasting Rice Genotypes
Source: PLoS One. 2012 Jun 28;7(6):e40203. doi: 10.1371/journal.pone.0040203 (PMC3386172; doi:10.1371/journal.pone.0040203)

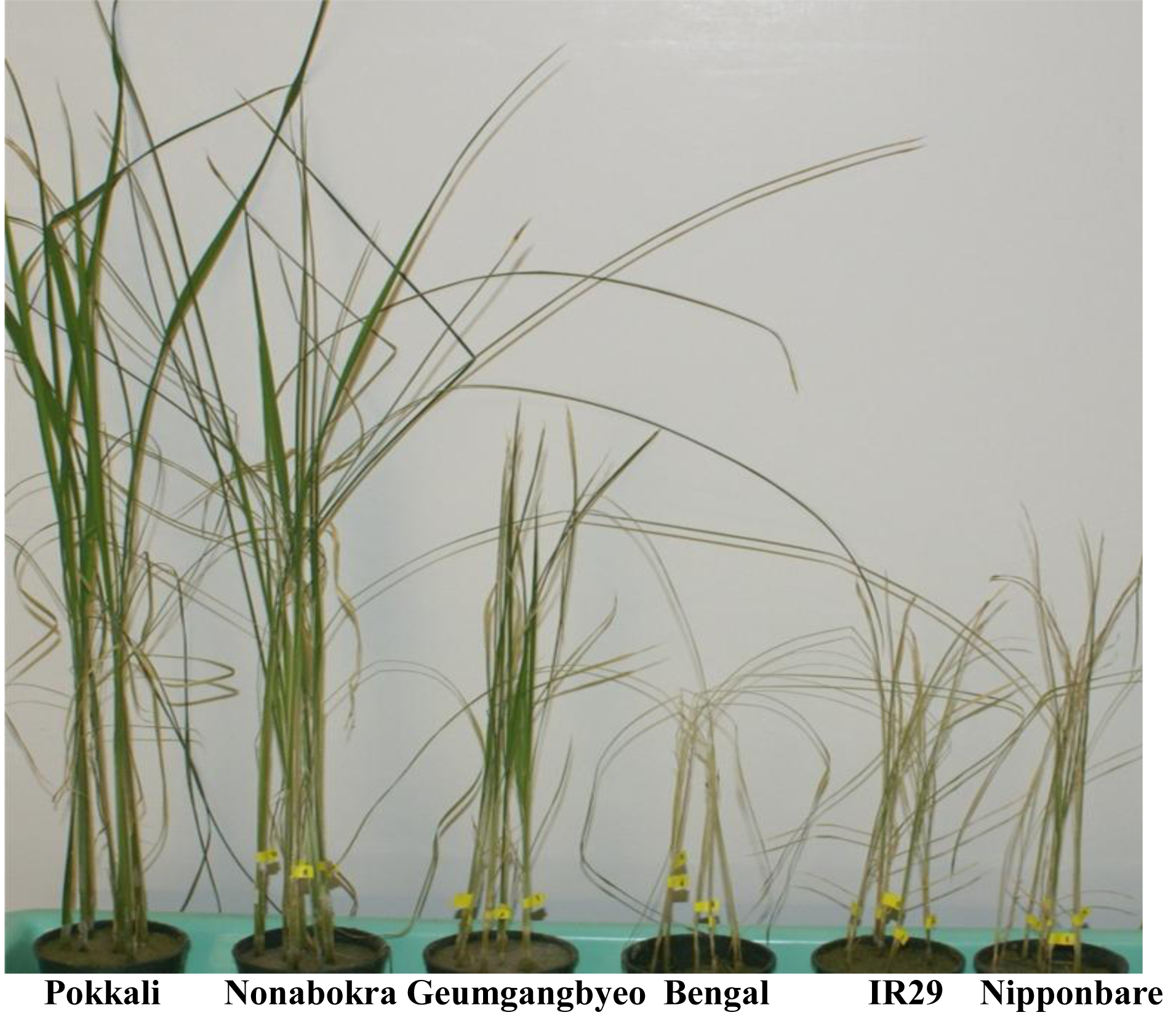

Supplement: Figure S1 — Phenotype of rice genotypes after 15 days of salinity stress (150 mM NaCl). Individual plants were scored for visual salt injury on a scale of 1–9 using the modified Standard Evaluation System [18]. (TIF) [file pone.0040203.s001.tif]

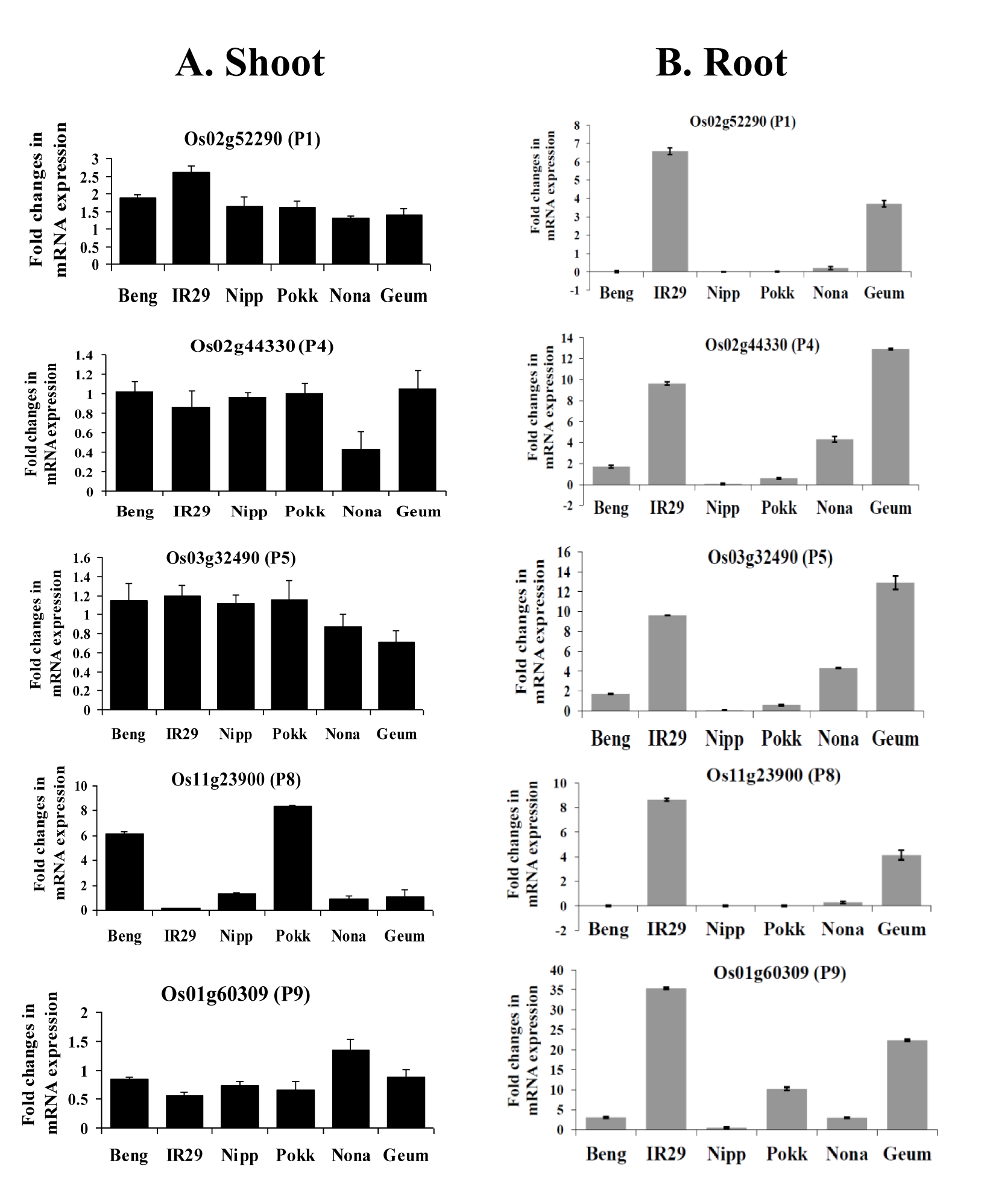

Supplement: Figure S2 — Effect of 150 mM NaCl on the K+/Na+ ratio in the shoot of six rice genotypes. The rice genotypes are Bengal (Beng), IR29, Nipponbare (Nipp), Pokkali (Pokk), Nonabokra (Nona), and Geumgangbyeo (Geum). Each value is the mean ± standard error from three independent replicates after exposure of 15 day old seedlings to salt stress for 7 days. (TIF) [file pone.0040203.s002.tif]

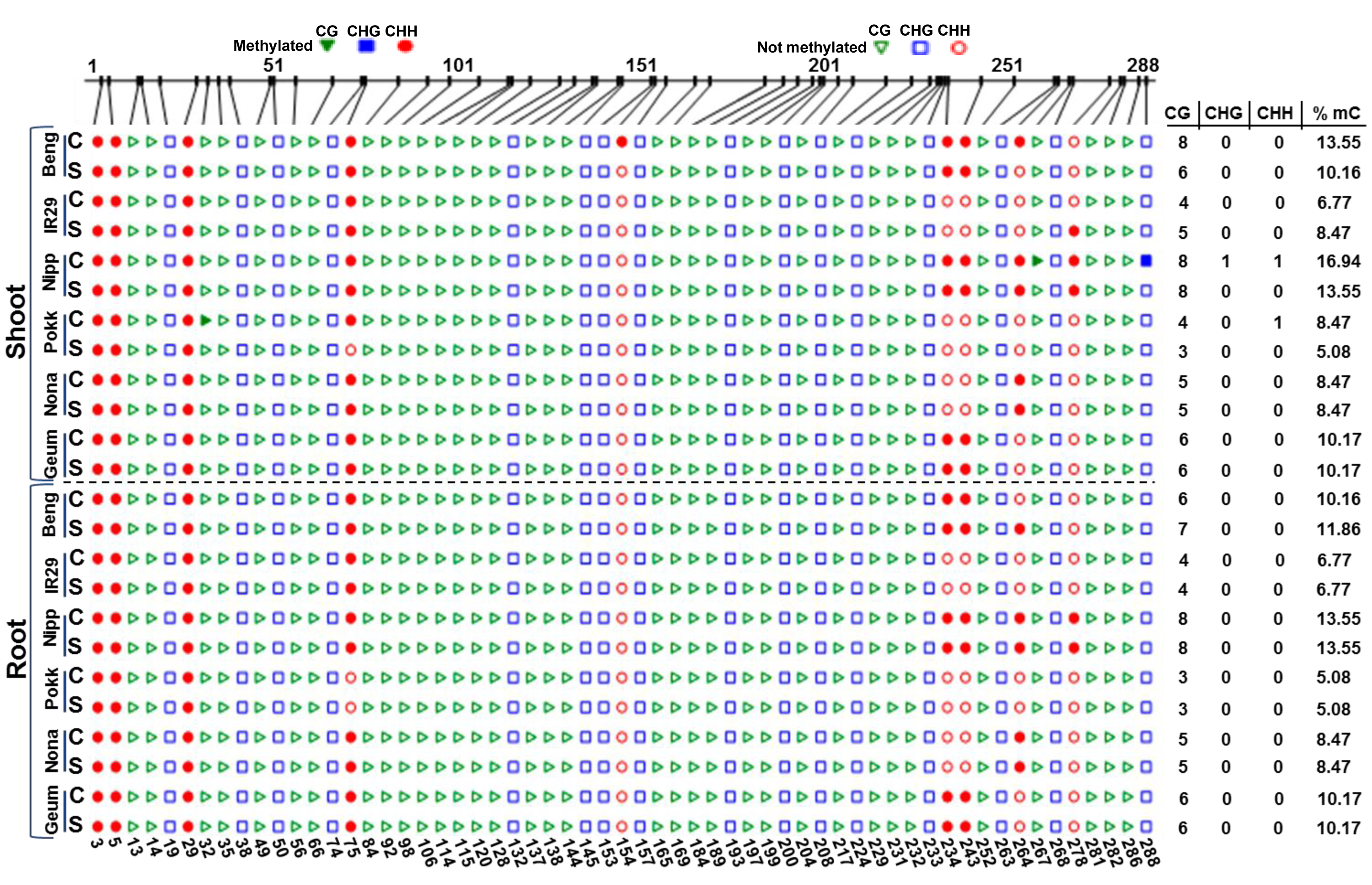

Supplement: Figure S3 — Bisulfite sequencing analysis of the locus Os02g52290 (P1). Top strand of Os02g52290 was sequenced from the shoot and root of six genotypes of rice, Bengal (Beng), IR29, Nipponbare (Nipp), Pokkali (Pokk), Nonabokra (Nona), and Geumgangbyeo (Geum) under non-stress and salinity stress (150 mM NaCl for 24 h). Cytosine methylation count was given for three types of methylation, CH, CHG, and CHH (H: A, T or C). C: non-stress; S: 150 mM salinity stress; % mC: percentage of methylated cytosines. (TIF) [file pone.0040203.s003.tif]

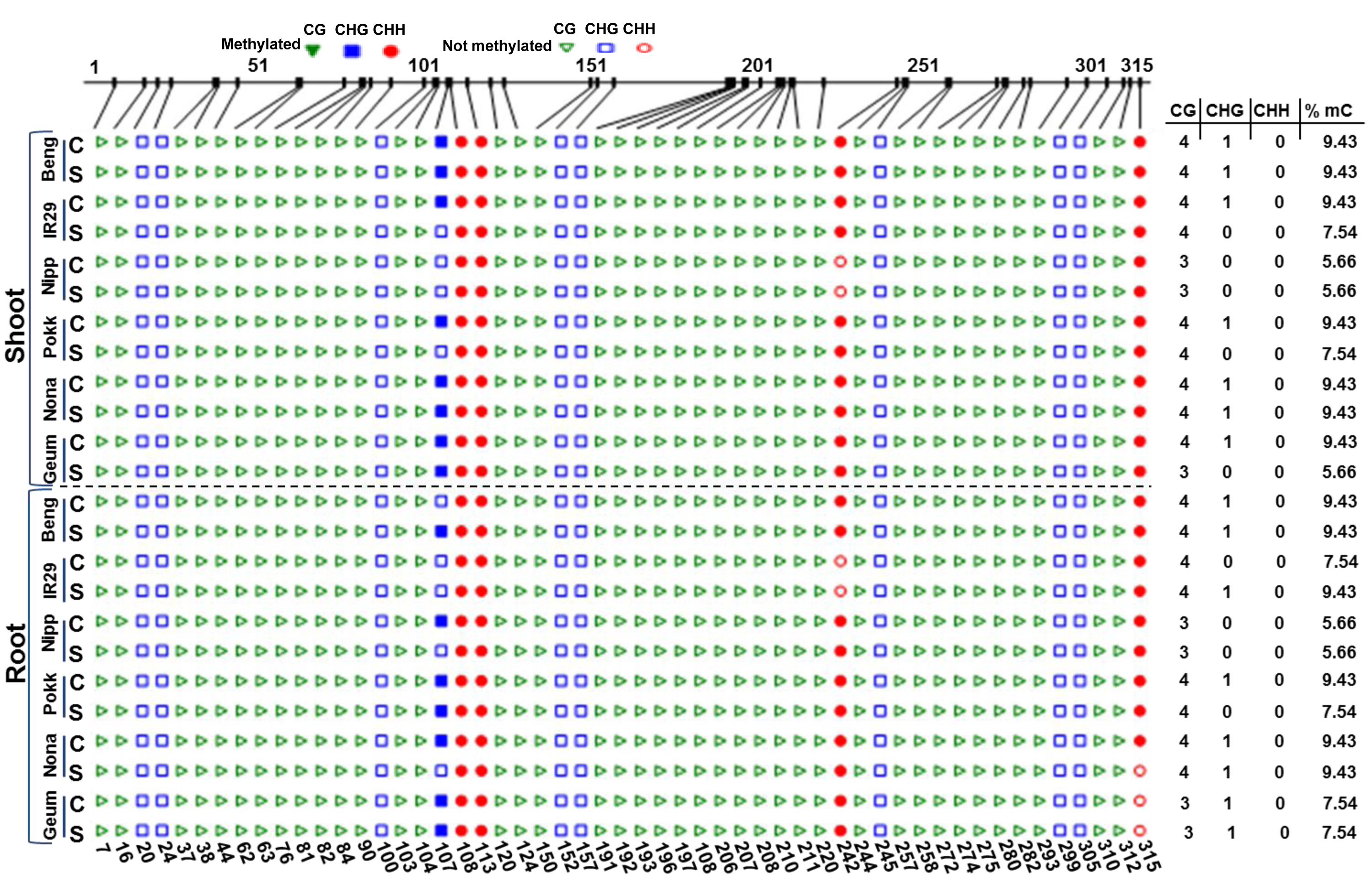

Supplement: Figure S4 — Bisulfite sequencing analysis of the locus Os01g60309 (P9). Top strand of Os01g60309 was sequenced from the shoot and root of six genotypes of rice, Bengal (Beng), IR29, Nipponbare (Nipp), Pokkali (Pokk), Nonabokra (Nona), and Geumgangbyeo (Geum), under non-stress and salinity stress (150 mM NaCl for 24 h). Cytosine methylation count was given for three types of methylation, CH, CHG, and CHH (H: A, T or C). C: non-stress; S: 150 mM salinity stress; % mC: percentage of methylated cytosines. (TIF) [file pone.0040203.s004.tif]

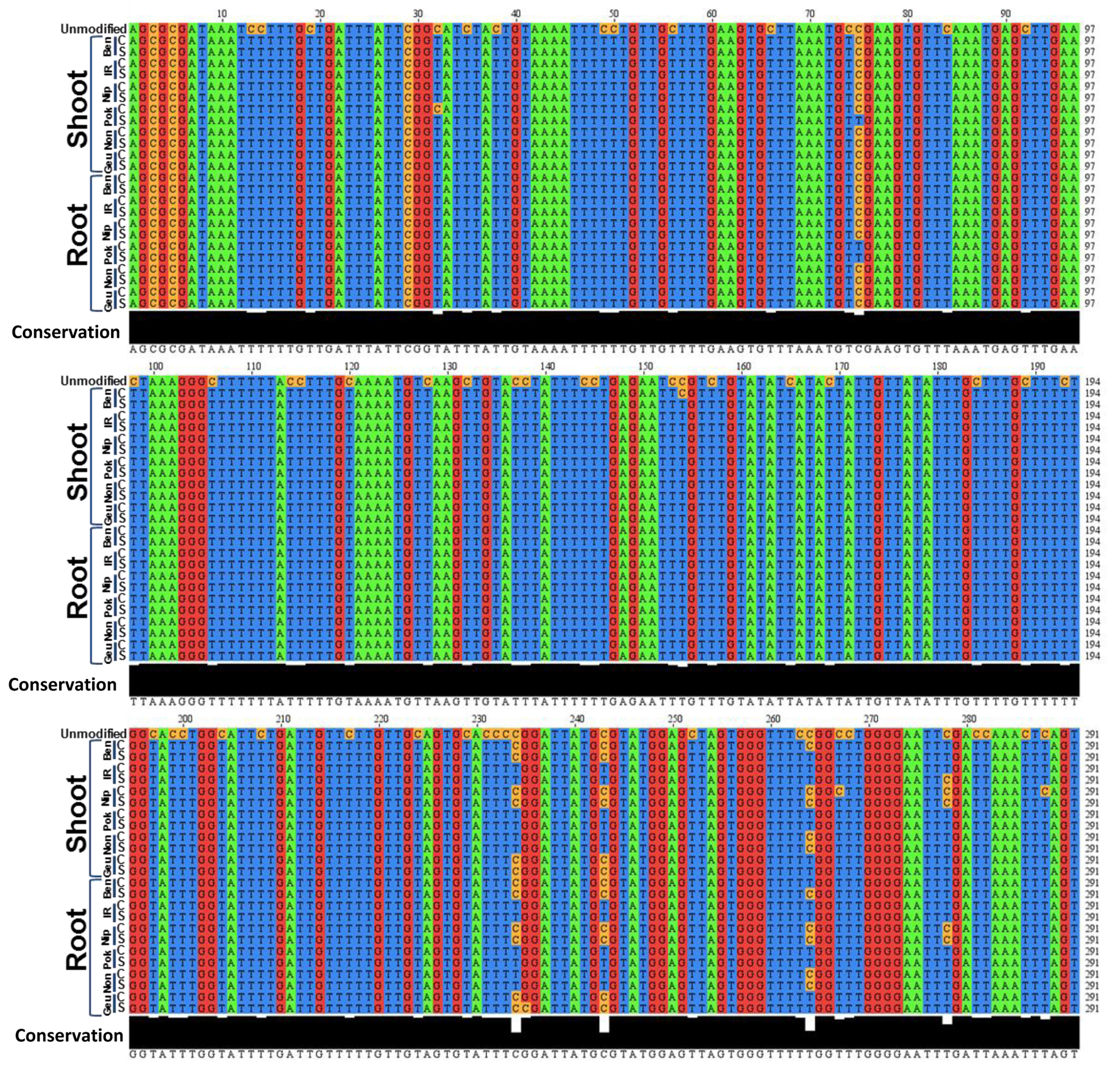

Supplement: Figure S5 — Sequence alignment of sodium-bisulfite-modified DNA of the locus Os02g52290 (P1). Top strand of Os02g52290 was sequenced from the shoot and root of six genotypes of rice, Bengal (Beng), IR29, Nipponbare (Nipp), Pokkali (Pokk), Nonabokra (Nona), and Geumgangbyeo (Geum) under non-stress and salinity stress (150 mM NaCl for 24 h. Unmodified represents top strand of unmodified sequence of Os02g52290 from Nipoonbare. C: non-stress; S: salinity stress. (TIF) [file pone.0040203.s005.tif]

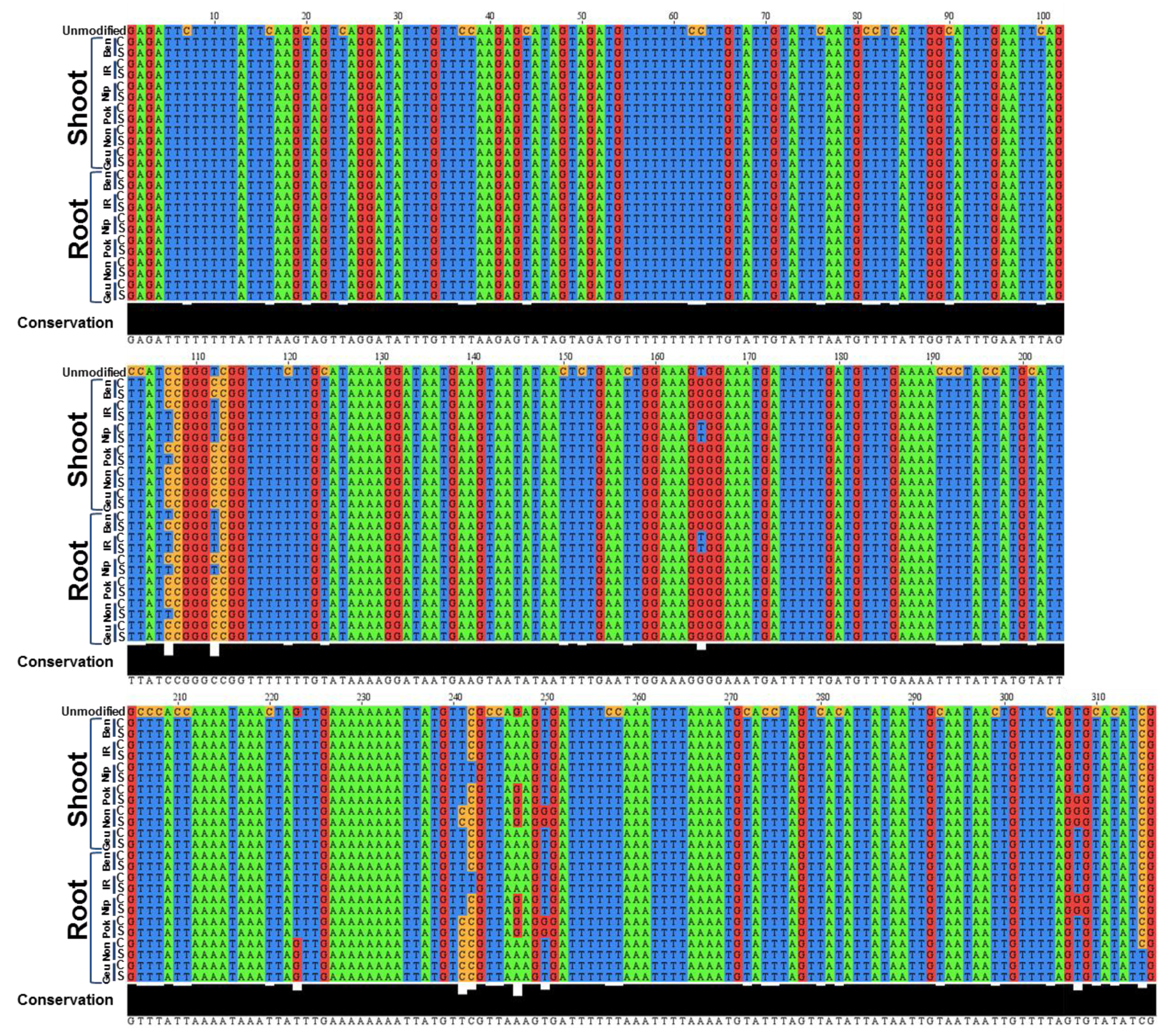

Supplement: Figure S6 — Sequence alignment of sodium-bisulfite-modified DNA of the locus Os01g60309 (P9). Top strand of Os02g52290 was sequenced from the shoot and root of six genotypes of rice, Bengal (Beng), IR29, Nipponbare (Nipp), Pokkali (Pokk), Nonabokra (Nona), and Geumgangbyeo (Geum) under non-stress and salinity stress (150 mM NaCl for 24 h). Unmodified represents top strand of unmodified sequence of Os01g60309 from Nipoonbare. C: non-stress; S: salinity stress. (TIF) [file pone.0040203.s006.tif]
