# Supplementary material for: Salt Stress Induced Variation in DNA Methylation Pattern and Its Influence on Gene Expression in Contrasting Rice Genotypes
Source: PLoS One. 2012 Jun 28;7(6):e40203. doi: 10.1371/journal.pone.0040203 (PMC3386172; doi:10.1371/journal.pone.0040203)
Supplement: Table S1 — Chi-square test and adjusted residuals for testing independence between methylation level and salt stress condition. (DOCX) [file pone.0040203.s007.docx]

Table S1: Chi-square test and adjusted residuals for testing independence between methylation level and salt stress condition.

| a. Shoot | | | | | | | | | |
| --- | --- | --- | --- | --- | --- | --- | --- | --- | --- |
| Treatment | Methylation Level | IR29 | | Nipponbare | | Pokkali | | Geumgangbyeo | |
|  |  | Number of observed loci | Adjusted residuals | Number of observed loci | Adjusted residuals | Number of observed loci | Adjusted residuals | Number of observed loci | Adjusted residuals |
| Control | Unmethylation | 665 | -4.35* | 877.00 | 6.49* | 698.00 | 4.75* | 838.00 | -8.16* |
| Control | Hemi-Methylatin | 201 | 5.23* | 246.00 | -2.07* | 293.00 | -1.07 | 143.00 | 1.59 |
| Control | Full-Methylation | 768 | 1.24 | 511.00 | -5.17* | 642.00 | -3.84* | 649.00 | 7.63* |
| Saline | Unmethylation | 789 | 4.35* | 692.00 | -6.49* | 566.00 | -4.75* | 1067.00 | 8.16* |
| Saline | Hemi-Methylation | 113 | -5.23* | 290.00 | 2.07* | 317.00 | 1.07 | 119.00 | -1.54 |
| Saline | Full-Methylation | 733 | -1.24 | 653.00 | 5.17* | 751.00 | 3.84* | 443.00 | -7.63* |
|  |  |  |  |  |  |  |  |  |  |
|  | Test Statistic | X^2^ | G^2^ | X^2^ | G^2^ | X^2^ | G^2^ | X^2^ | G^2^ |
|  |  | 36.05 | 36.39 | 42.75 | 42.85 | 23.26 | 23.29 | 68.59 | 68.9 |
|  | P- Value | <0.0001 | <0.0001 | <0.0001 | <0.0001 | <0.0001 | <0.0001 | <0.0001 | <0.0001 |
|  | DF | 2 | 2 | 2 | 2 | 2 | 2 | 2 | 2 |

| b. Root | | | | | | | | | |
| --- | --- | --- | --- | --- | --- | --- | --- | --- | --- |
| Treatment | Methylation Level | IR29 | | Nipponbare | | Pokkali | | Geumgangbyeo | |
|  |  | Number of observed loci | Adjusted residuals | Number of observed loci | Adjusted residuals | Number of observed loci | Adjusted residuals | Number of observed loci | Adjusted residuals |
| Control | Unmethylation | 1276 | -1.74 | 1103 | -8.23* | 1231 | -0.17 | 1311 | -0.42 |
| Control | Hemi-Methylatin | 7 | -2.94* | 34 | -0.59 | 32 | -0.26 | 10 | -1.68 |
| Control | Full-Methylation | 352 | 2.48* | 498 | 8.68* | 323 | 0.27 | 313 | 0.84 |
| Saline | Unmethylation | 1314 | 1.74 | 1310 | 8.23* | 1232 | 0.17 | 1319 | 0.42 |
| Saline | Hemi-Methylatin | 23 | 2.94* | 39 | 0.59 | 34 | 0.26 | 19 | 1.68 |
| Saline | Full-Methylation | 295 | -2.48* | 286 | -8.68* | 316 | -0.27 | 294 | -0.84 |
|  |  |  |  |  |  |  |  |  |  |
|  | Test Statistic | X^2^ | G^2^ | X^2^ | G^2^ | X^2^ | G^2^ | X^2^ | G^2^ |
|  |  | 14.11 | 14.57 | 75.43 | 76.17 | 0.13 | 0.13 | 3.41 | 3.46 |
|  | P- Value | 0.0009 | 0.0007 | <0.0001 | <0.0001 | 0.9358 | 0.9358 | 0.1817 | 0.1775 |
|  | DF | 2 | 2 | 2 | 2 | 2 | 2 | 2 | 2 |
