# Supplementary material for: Salt Stress Induced Variation in DNA Methylation Pattern and Its Influence on Gene Expression in Contrasting Rice Genotypes
Source: PLoS One. 2012 Jun 28;7(6):e40203. doi: 10.1371/journal.pone.0040203 (PMC3386172; doi:10.1371/journal.pone.0040203)
Supplement: Table S2 — Adapters and primers sequences used for methylation sensitive amplified polymorphism analysis. (DOCX) [file pone.0040203.s008.docx]

Table S2 Adapters and primer sequences used for methylation sensitive AFLP.

| Adapter and Primer | *EcoR*I (5’-3’) | *Hpa*II */ Msp*I (5’-3’) |
| --- | --- | --- |
| Adapter 1 | CTCGTAGACTGCGTACC | GATCATGAGTCCTGCT |
| Adapter 2 | AATTGGTACGCAGTCTAC | CGAGCAGGACTCATGA |
| Preamplification primer | GACTGCGTACCAATTC (E) | ATCATGAGTCCTGCTCGG (HM) |
| Selective amplification primer | E+AAG_700 | HM+AT |
|  | E+AAC_700 | HM+AG |
|  | E+ACG_800 | HM+AC |
|  | E+AGC_800 | HM+AAT |
|  |  | HM+ACT |
|  |  | HM+ATC |
|  |  | HM+TCAA |
|  |  | HM+AATC |

Note: Numerals 700 and 800 denotes labeling of *EcoR*I primers with fluorescent dyes amino C6-DY682 and amino C6-DY782 respectively.
