# Supplementary material for: Salt Stress Induced Variation in DNA Methylation Pattern and Its Influence on Gene Expression in Contrasting Rice Genotypes
Source: PLoS One. 2012 Jun 28;7(6):e40203. doi: 10.1371/journal.pone.0040203 (PMC3386172; doi:10.1371/journal.pone.0040203)
Supplement: Table S3 — Primers used for quantitative reverse transcription polymerase chain reaction. (DOCX) [file pone.0040203.s009.docx]

Table S3 Primers used for quantitative reverse transcription polymerase chain reaction.

| Gene | Forward (5’-3’) | Reverse (5’-3’) |
| --- | --- | --- |
| Os11g23900 | TGGCGAGGCGGTTTTACCACAC | AAGTCGCGGCAACTAAGGCGG |
| Os2g44330 | GGCACCAGAAGAGGAGTCAC | TGAGGTCAACTTCTCCACCA |
| Os3g32490 | GTCGAGGACTTCCTGCACTC | AGTAGCGGTCCCTGCAGTAG |
| Os2g52290 | TCAATATAGGCCAGGGTTCG | TACTGGGCGCTAAGAACCTC |
| Os01g60309 | TCTGGAGATTCGAACGGAAC | TGATCAAACACTGGGCTCTG |
| *eEF-1*alpha | AGGTTGCCTGAGTCACAGTTAAGTG | TTTCACTCTTGGTGTGAAGCAGAT |
